# An Amperometric Biosensor Utilizing a Ferrocene-Mediated Horseradish Peroxidase Reaction for the Determination of Capsaicin (Chili Hotness)

**DOI:** 10.3390/s130810014

**Published:** 2013-08-05

**Authors:** Rosmawani Mohammad, Musa Ahmad, Lee Yook Heng

**Affiliations:** School of Chemical Sciences and Food Technology, Faculty of Science and Technology, Universiti Kebangsaan Malaysia (UKM), 43600 Bangi, Selangor Darul Ehsan, Malaysia; E-Mails: rosaz99@yahoo.com (R.M.); andong@ukm.my (M.A.)

**Keywords:** capsaicin, chili hotness, ferrocene, horseradish peroxidase, photocured membrane

## Abstract

Chili hotness is very much dependent on the concentration of capsaicin present in the chili fruit. A new biosensor based on a horseradish peroxidase enzyme-capsaicin reaction mediated by ferrocene has been successfully developed for the amperometric determination of chili hotness. The amperometric biosensor is fabricated based on a single-step immobilization of both ferrocene and horseradish peroxidase in a photocurable hydrogel membrane, poly(2-hydroxyethyl methacrylate). With mediation by ferrocene, the biosensor could measure capsaicin concentrations at a potential 0.22 V (*vs*. Ag/AgCl), which prevented potential interference from other electroactive species in the sample. Thus a good selectivity towards capsaicin was demonstrated. The linear response range of the biosensor towards capsaicin was from 2.5–99.0 μM with detection limit of 1.94 μM. A good relative standard deviation (RSD) for reproducibility of 6.4%–9.9% was obtained. The capsaicin biosensor demonstrated long-term stability for up to seven months. The performance of the biosensor has been validated using a standard method for the analysis of capsaicin based on HPLC.

## 1. Introduction

Chili is the fruit of the plants from the genus *Capsicum*. Chili has been employed in a variety of folk remedies to treat conditions such as asthma, lumbago, neuralgia, pneumonia, rheumatism, sores, cancers and tumors [1]. It is also rich in vitamin C, as an antioxidant and red chili is a good source of β-carotene. However, chili is better known for its pungency and is widely used as a spice in many cuisines. All hot chili fruits contain capsaicinoids, natural substances responsible for the hotness and pungent taste of chili. Capsaicin and dihydrocapsaicin comprise 80%–90% of the capsaicinoids found in chili [2]. Capsaicin is the main capsaicinoid in chili.

For a few decades, amperometric enzyme-based biosensors have grown rapidly as an analytical tool applied in various fields such as environmental monitoring, biotechnology, clinical analysis, food and agricultural product processing. This is due to their good selectivity, sensitivity, miniature size, fast response and reproducible results [3,4]. Amperometric enzyme-based biosensors can be developed for the determination of pungency level of chili in addition to the existing Scoville method and chromatographic techniques. The main advantages of biosensor technique is the need of only minimal sample pre-treatment and the portable capability for performing on-site analysis. To date, the only capsaicin sensor reported was based on stripping voltammetry using multi-walled carbon nanotube modified screen-printed electrode and graphite electrode [5]. The operating potentials of these electrochemical sensors were 0.48 & 0.70 V (*vs*. SCE) and this caused the sensor to become vulnerable to many interference substances. To prevent the use of high operating potential, we have recently explored a new mediated enzyme reaction of horseradish peroxidase (HRP) for the determination of capsaicin.

HRP is a heme-containing glycoprotein [6] that catalyses the oxidation of aromatic compounds in the presence of hydrogen peroxide (H_2_O_2_) [7]. However, the direct electrochemistry of HRP is complicated [6]. Peroxidase catalysis is associated with four types of activity *i.e*., peroxidative, oxidative, catalytic and hydroxylation. Under the usual assay conditions where a phenolic substrate is used, the reaction involved is peroxidative reaction. Basically peroxidase catalyses a reaction in which hydrogen peroxide acts as the acceptor and the phenolic compounds, acts as the donor of hydrogen atoms. In this case molecular oxygen is not a product of the reaction [8].

**Figure 1.**
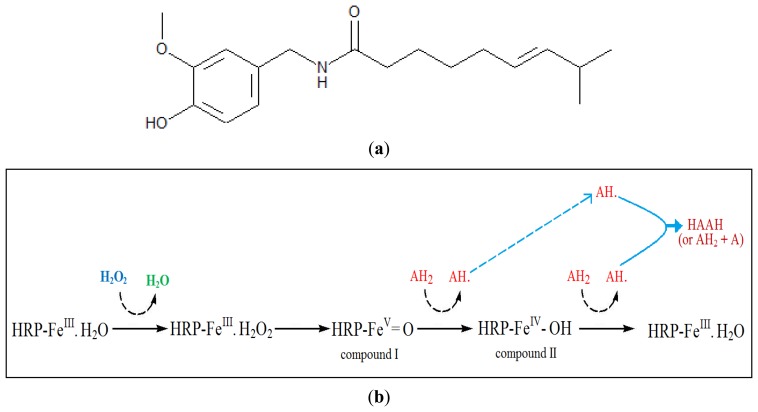
The structure of capsaicin (a); and the proposed reaction mechanism of peroxidase with capsaicin and hydrogen peroxide (b). AH_2_ & AH• = molecule & free radical from the hydrogen donor (capsaisin or phenolic compounds); HRP = Horseradish peroxidase; HAAH = polymerized product.

Capsaicin also behaves as a phenolic compound because of the phenol moiety in its structure (Figure 1a). Therefore, the mechanism for the enzymatic reaction of HRP and capsaicin could be explained by phenolic compound reaction with HRP in the presence of hydrogen peroxide. The mechanism reaction of HRP and phenolic compound in the presence of hydrogen peroxide has been explained detail by Whitaker [8]. The suggested reaction mechanism of peroxidase with capsaicin and hydrogen peroxide (modified from [8]) was shown in Figure 1b.

Horseradish peroxidase contains one ferriprotoporphyrin III (protohemin) group per molecule. In the first step hydrogen peroxide replaces water at one of the iron of the protohemin to form enzyme-substrate complex (HRP-Fe^III^·H_2_O_2_). The second step involves the formation of compound I (HRP–Fe^V^=O). In the third step, compound I reacts with an exogenous donor (AH_2_) if available (such as capsaicin), to give compound II (HRP–Fe^IV^–OH) and a free radical from the hydrogen donor (AH•). In the fourth step, compound II reacts with a second molecule of the hydrogen donor (AH_2_) to regenerate the original enzyme (HRP–Fe^III^·H_2_O) and a free radical from the hydrogen donor (AH•). The free radicals interact with each other to give a polymerized product shown as HAAH [8].

The enzyme HRP has been used for determination of phenolic compounds based on amperometric biosensors [9–11]. Biosensor-based HRP enzyme shows a response mechanism to phenolic compounds called double displacement or ping-pong mechanism, in which two substrates, the hydrogen peroxide and the electron-donating phenolic-derived compound are involved. At the electrode surface, the enzymes molecules are oxidized by hydrogen peroxide followed of its reduction by the phenolic compound [9]. The sensitivity of these peroxidase-based biosensors is limited by high current due to direct electron transfer between the enzyme and the electrode surface in the presence of hydrogen peroxide [9].

**Scheme 1.**
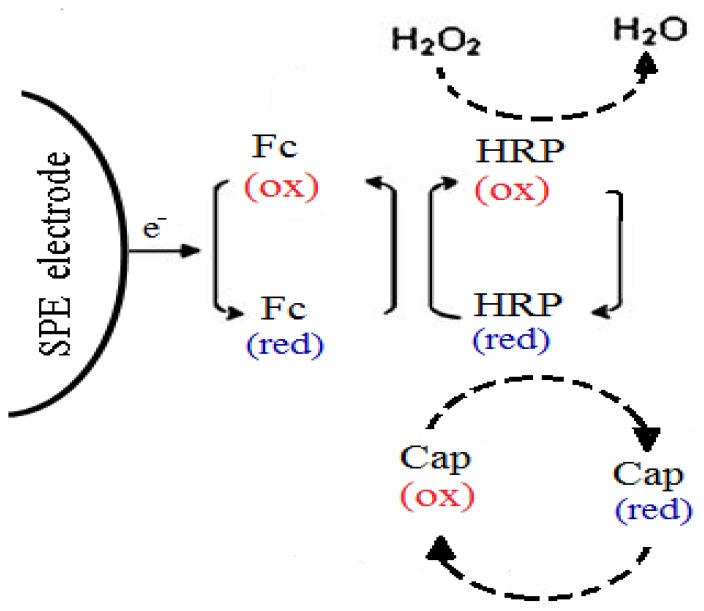
The principle of ferrocene mediated reaction of HRP-capsaicin-H_2_O_2_ that occurs at the surface of the screen-printed carbon-paste electrode. SPE = screen-printed carbon paste electrode; Fc = ferrocene; HRP = horseradish peroxidase; Cap = capsaicin; ox = oxidation state; red = reduction state.

In this study, to enable a lower operating potential of the capsaicin biosensor based on HRP, ferrocene is used as a redox mediator to shuttle electrons between the electrode and enzyme active site [11–13]. The use of ferrocene in the amperometric biosensor allows reversible redox reactions [14] and it has been used in this manner for many amperometric biosensors for the determination of phenolic acids with other enzymes [12,15]. Thus, Sulak *et al*. [11] used ferrocene as a mediator for the development of an amperometric phenol HRP enzyme-based biosensor. In this study, this proposed mechanism is utilized for the direct analysis of capsaicin by designing an amperometric biosensor. The principle reaction between HRP and capsaicin in the presence of H_2_O_2_ that was mediated by ferrocene in the biosensor membrane system is given in Scheme 1. The biosensor was fabricated via a single-step procedure by incorporating both ferrocene and horseradish peroxidase in a photocured 2-hydroxyethyl methacrylate membrane [16].

## 2. Experimental Section

### 2.1. Reagents

Horseradish peroxidase type VI-A (EC 1.11.1.7), 2-hyroxyethyl methacrylate (HEMA) monomer and catechol 99% were purchased from Sigma (St. Louis, MO, USA). The photoinitiator 2,2-dimethoxy-2-phenylacetophenone (DMPP), ferrocene, hydrogen peroxide and capsaicin, were obtained from Fluka (Buchs, Switzerland). Other chemicals that have been used were acid acetic glacial (Ajax Chemicals, Bankstown, Australia), resorcinol (Mallinckrodt, MO, USA), phenol (Panreac, Barcelona, Spain) and guaiacol/2-methoxyphenol (Trade TCI, Tokyo, Japan). Potassium dihydrogen phosphate, dipotassium hydrogen phosphate, sodium acetate trihydrate and sodium chloride were from Merck (Darmstadt, Germany). 2,4-Dimethylphenol 99%, 3-chlorophenol 99%, 4-chloro-3-methylphenol 99%, 3,4-dimethylphenol 99% and 2-aminophenol 99% were from Acros Organics (New Jersey, NJ, USA).

Solutions stock of 0.01 M capsaicin was prepared in 95% ethanol. Nine types of phenolic compounds at 0.01 M concentration were also prepared in 95% ethanol. These phenolic compounds were catechol, phenol, guaiacol, 2.4-dimethylphenol, 3-chlorophenol, 3,4-dimethylphenol, 2-aminophenol, 4-chloro-3-methylphenol and resorcinol.

Solution stock for 1 M H_2_O_2_ was prepared in deionized water. HRP enzyme was weighed and dissolved in phosphate buffer pH 6. Phosphate buffer 0.1 M at pH 7 was prepared from potassium dihydrogen phosphate and dipotassium hydrogen phosphate. 0.1 M NaCl was prepared by dissolving NaCl in 0.1 M phosphate buffer pH 7.

### 2.2. Fabrication of Capsaicin Biosensor

Poly-2-hydroxyethyl methacrylate membrane containing HRP and ferrocene was prepared as reported by Bean *et al*. [17]. A mixture of 2-hydroxyethyl methacrylate monomer (5 mL) containing ferrocene and HRP enzyme at 1:1 ratios were deposited on screen-printed carbon-paste electrodes (SPE). This was then exposed to UV radiation under the nitrogen flow for 360 s to form a thin layer of membrane.

### 2.3. Amperometric Measurements

Amperometric measurements were carried out in a stirred electrochemical cell containing 4 mL of 0.1 M phosphate buffer at pH 7 and 0.1 M NaCl in the presence of 100 μM H_2_O_2_ using an Autolab PGSTAT 12 Potentiostat (Metrohm) (Utrecht, Netherlands). SPE coated with horseradish peroxidase/ferrocene containing photocured membrane was used as a working electrode, Ag/AgCl (3 M KCl) as a reference electrode and platinum (Metrohm) (Utrecht, Netherlands) as a counter electrode. The measurements of capsaicin were studied at fixed potential 0.22 V (*vs*. Ag/AgCl) under continuous stirring at 100 rpm by using chronoamperometry method. All measurements (current readings) were taken at 30 min unless otherwise stated and measured *versus* the Ag/AgCl reference electrode.

### 2.4. Interference Study of Biosensor

Interference study for capsaicin biosensor was carried out by using several phenolic compounds (e.g., catechol, phenol, guaiacol, 2.4-dimethylphenol, 3-chlorophenol, 3,4-dimethylphenol, 2-aminophenol, 4-chloro-3-methylphenol and resorcinol). The concentration of capsaicin used was 49.5 μM and 0.99 μM concentration of interfering compounds. Biosensor response towards capsaicin (49.5 μM) without the presence of interfering compounds also recorded to obtain the percentage of interference when the interfering substances were present.

### 2.5. Chili Sample Analysis

Chili samples were crushed using a mortar before extraction. The chili extraction was carried out by using two methods. First method is according to AOAC, Official Method of Analysis 995.03 [18]. In this method, chili (25 g) was extracted in ethanol (200 mL) for five hours using a reflux condenser. In the second method, the extraction was carried out at room temperature for 15 min using 25 g chili. For HPLC analysis, the extraction of chili was filtered using a special syringe filter with a membrane size 0.45 μm. Capsaicin analysis was performed using the HPLC method (AOAC Official Method 995.03 [18] with a C18 column, mobile phase acetonitrile-water containing 1% acetic acid (v/v), flow rate 1.5 mL/min and UV detector at wavelength 280 nm.

The biosensor response at different concentrations of capsaicin standard concentration was validated with the HPLC method. The recovery study was carried out by spiking capsaicin standards at different concentrations in chili samples. Chili samples without spiking with capsaicin standard were also analyzed for capsaicin. The percentage of recovery was calculated with consideration of the capsaicin content of the non-spiked samples [19].

### 2.6. Long Term Response of the Biosensor

In order to study the long term response of the biosensor, several biosensors were prepared and stored at 4 °C to be tested every month. Different biosensors were used for each measurement and amperometric measurements were performed at 99.0 μM capsaicin in the presence of 100 μM H_2_O_2_. The measurement was performed in triplicate.

## 3. Results and Discussion

### 3.1. Cyclic Voltammogram and the Response of Amperometric Capsaicin Biosensor

**Figure 2.**
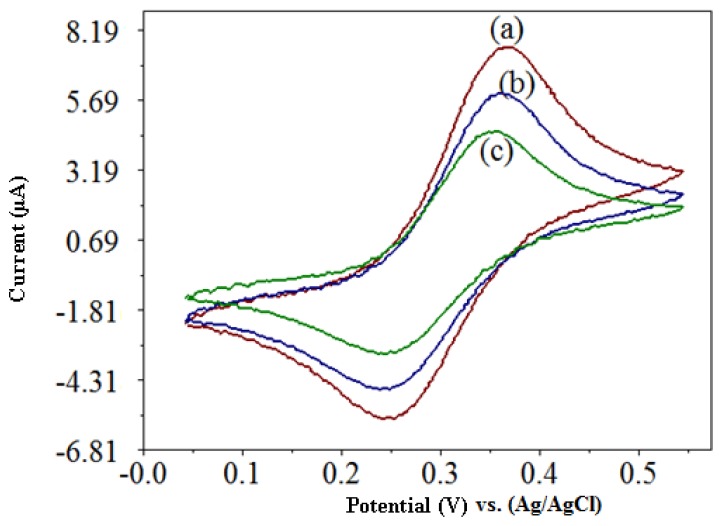
The cyclic voltammograms of the capsaicin biosensor with photocured membrane containing HRP-ferrocene in phosphate buffer pH 7/0.1 M NaCl (a); after addition of 123.5 μM H_2_O_2_ (b); followed by addition of 129.5 μM capsaicin (c).

The cyclic voltammetric response of the capsaicin and H_2_O_2_ are shown in Figure 2. In phosphate buffer pH 7/0.1 M NaCl alone, the biosensor exhibited the electrochemical behavior of the immobilized ferrocene, where the oxidation and reduction peaks are observed (Figure 2a). When 123.5 μM H_2_O_2_ was added to the buffer solution, the cathodic and anodic peak currents decreased (Figure 2b). This indicates that the reaction between HRP and H_2_O_2_ was mediated by the ferrocene, where the electrons are shuttled from the redox center of HRP to the electrode surface via redox reactions of ferrocene. Thus, ferrocene acts as an electron donor during the reaction and was oxidized, which leads to a decrease in current measured [12]. With the addition of 129.5 μM of capsaicin the cathodic and anodic peak current decreased further (Figure 2c). It showed that HRP enzyme was first oxidized by H_2_O_2_ and followed of its reduction by capsaicin [9,10].

**Figure 3.**
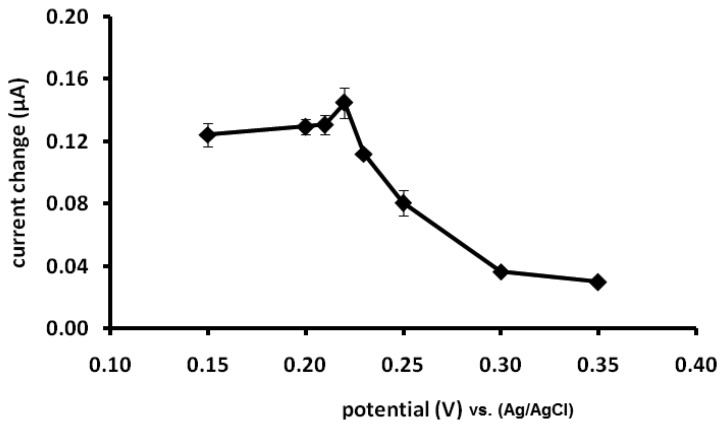
Effect of applied potential on the current response of the biosensor (measurement was performed at 196.1 μM capsaicin in the presence of 50 μM H_2_O_2_ in phosphate buffer pH 7/0.1 M NaCl).

**Figure 4.**
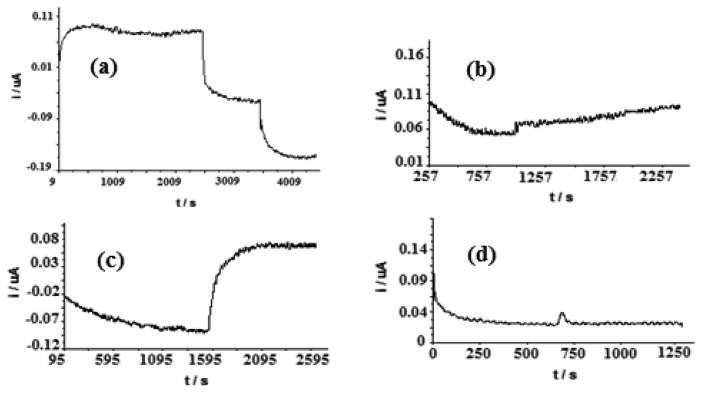
The current response of a biosensor: At 50 μM and 100 μM H_2_O_2_ (a); 196.1 μM capsaicin (b); 196.1 μM capsaicin in the presence of 50 μM H_2_O_2_ (c) and an electrode without HRP enzyme in 196.1 μM capsaicin and 50 μM H_2_O_2_ (d). All test solutions were in phosphate buffer pH 7/0.1 M NaCl.

From Figure 3, the current response of the biosensor was increased with increasing applied potentials from 0.15 to 0.22 V. Beyond 0.22 V, the current decreased and therefore 0.22 V was chosen as the optimized potential for further amperometric measurements. The responses of the biosensor to added capsaicin only, H_2_O_2_ only and capsaicin in the presence of H_2_O_2_ were different (Figure 4). The response towards H_2_O_2_ was opposite to that of capsaicin (Figure 4a). This is because in the absence of hydrogen donor, peroxidase behaves as a catalase converting H_2_O_2_ to water and oxygen [8].

At capsaicin concentration of 196.1 μM alone, the current response increased by 35.9 ± 1.3 nA (Figure 4b). While at the same concentration of capsaicin in the presence of H_2_O_2_, the current increase was enhanced (156.2 ± 4.6 nA) (Figure 4c). As reported by Pomar *et al*. [7], peroxidase could oxidize capsaicinoids, however it will react more quickly in the presence of H_2_O_2_. For an electrode without immobilized HRP in the presence of 196.1 μM capsaicin and 50 μM H_2_O_2_, no current change could be observed (Figure 4d).

### 3.2. The Optimized Conditions for the Capsaicin Biosensor Response

The response of the biosensor for the determination of capsaicin appeared to be best at pH 7.0 and thus this pH was chosen for all studies. A similar observation was reported by Wang *et al*. [15] when they used HRP enzyme for the measurement of hydrogen peroxide concentration with amperometric method. The effect of ferrocene concentration immobilized on the biosensor response to capsaicin was studied by varying the amount of ferrocene immobilized in the photocured membrane. The best current response was obtained at 1 wt% of ferrocene. The enzyme loading can also influence the response of the biosensor towards capsaicin. For the range of 0–21 units of immobilized HRP, the current increased steadily until saturation values after 14 units of HRP was incorporated. Thus, the optimum enzyme loading for the biosensor was 14 units. The study of the effect of H_2_O_2_ concentration is very important because HRP reaction with phenolic compounds is hydrogen peroxide dependent [11] and it will affect the sensitivity of the biosensor [9]. The optimum concentration of H_2_O_2_ that yielded optimum current in this study was 100 μM.

### 3.3. Amperometric Capsaicin Biosensor Performance

**Figure 5.**
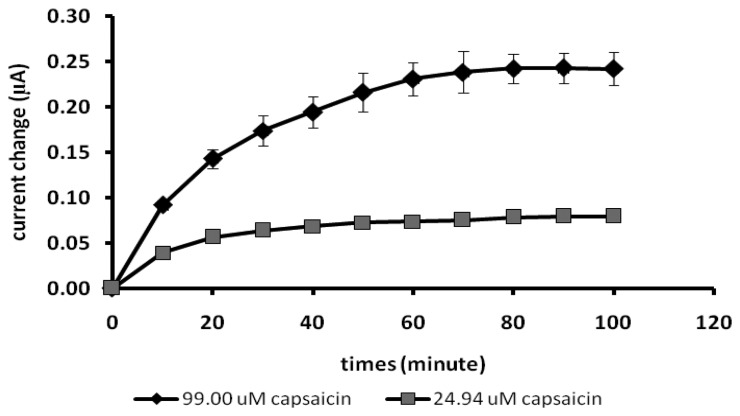
The response time of the capsaicin biosensor at 24.94 and 99.0 μM capsaicin concentrations (test solution: phosphate buffer pH 7/0.1 M NaCl and 100 μM H_2_O_2_).

By using the optimized conditions mentioned above, the current responses of the capsaicin biosensor to certain fixed concentrations of capsaicin with time are shown in Figure 5. A steady state current can be achieved after 50 min of exposure of the biosensor to capsaicin. This long response time is due to the slow diffusion of the analyte into the photocured membrane as has been observed before [20].

**Figure 6.**
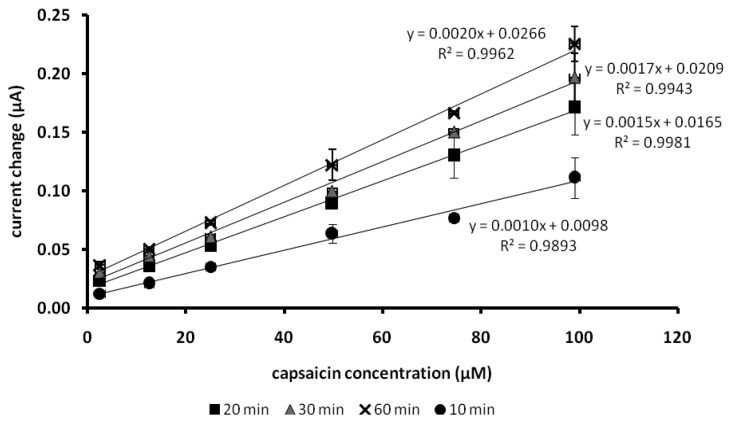
The biosensor responses taken at different exposure times towards various capsaicin concentrations (Test solution: phosphate buffer pH 7/0.1 M NaCl. H_2_O_2_ 100 μM).

As mentioned by Ahmad and Narayanaswamy [21], a kinetic approach can be used for analysis without have to wait for the steady state response of the sensor to be achieved. Although the capsaicin biosensor needed a long response time to achieve a steady state response, as depicted in Figure 6, the responses of the capsaicin biosensor towards increasing concentrations of capsaicin recorded at times after 20 min showed similar trend, linearity and response slope.

**Figure 7.**
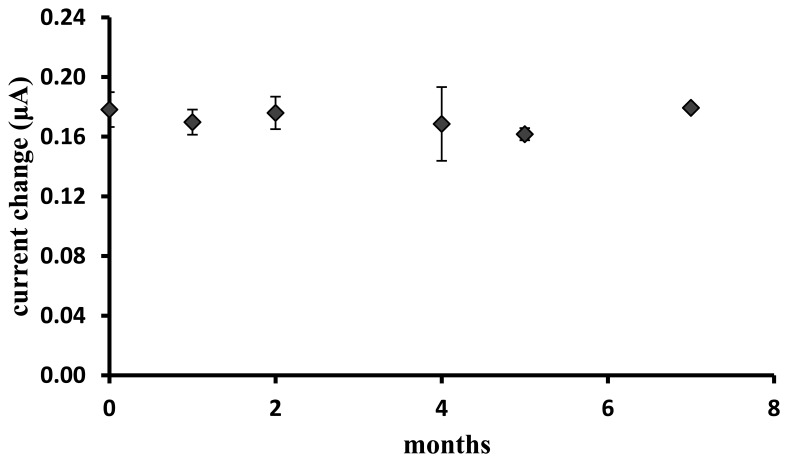
The effect of storage time on the stability of the capsaicin biosensor response measured at 99.0 μM capsaicin (100 μM H_2_O_2_ and phosphate buffer pH 7/0.1 M NaCl).

Using current data obtained at four different response times, *i.e*., 10–60 min, the reproducibility of the biosensor at capsaicin concentration of 99.0 μM gave good relative standard deviations (RSD) in the range of 6.4%–9.9%. As depicted in Figure 7, the biosensor response still retained up to 95% of its original value after seven months of storage at 4 °C. This long-term stability behaviour was due to the ability of the photocured hydrophilic membrane to retain water and hence maintaining the enzyme activity [22].

### 3.4. Validation and Recovery Study

**Figure 8.**
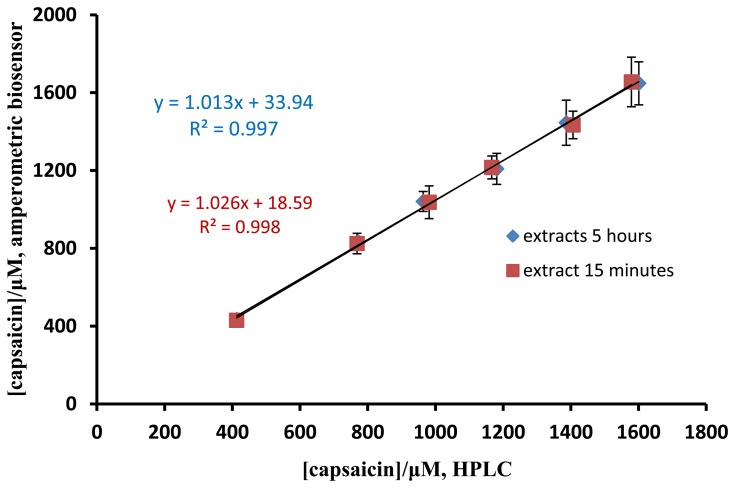
A comparison between the capsaicin biosensor and HPLC method for the determination of capsaicin.

The pungency level of a chili sample can be evaluated via the capsaicin concentration. The performance of the capsaicin biosensor developed here was compared with standard method for capsaicin analysis by AOAC method using HPLC [18]. Chili samples spiked with different concentrations of capsaicin standard were used for the validation study. For the extraction of chili samples, the duration was carried out at 15 min and 5 h. The results of capsaicin determined by both methods were not significant difference and a good correlation between the two methods was obtained (Figure 8). Furthermore, the duration of extraction did not affect the capsaicin concentration determined. As shown in Table 1, a good recovery (96.8%–102.0%) was obtained for the determination of spiked capsaicin by the biosensor.

Based on the results reported by Schweiggert *et al*. [23] on the content of capsaicinoids and related compounds in chili pods (*Capsicum frutescens* L.) determined by high-performance liquid chromatography/atmospheric pressure chemical ionization mass spectrometry, capsaicinoids were the predominant phenolic components in chili whilst other types of phenolic compounds present only in small quantities, the good percentage of recovery using the biosensor developed here has confirmed such observation, *i.e*., the absent of major interferences.

### 3.5. Comparison with Reported Amperometric Determination of Capsaicin

To date only one sensor that based on amperometric measurement has been reported [5]. Based on Figure 6, the linearity of the capsaicin biosensor towards capsaicin reported here was in the concentration range of 2.5–99.0 μM with detection limit of 1.94 μM. Kachoosangi *et al*. [5] reported that the electrochemical capsaicin sensors with the linearity of capsaicin concentration in the range of 0.5–35.0 μM and detection limit of 0.31–0.45 μM. The amperometric biosensor in this work clearly demonstrated a much wider linear response range. The LOD obtained is below the limit of total capsaicinoids in foods recommended by the Council of Europe [24] for foods and beverages (<5 ppm). This is an improvement when compared with other reported electrochemical sensors for capsaicin that operated at much higher potentials of 0.70 V and 0.48 V (*vs*. SCE). At such potentials, the sensor is vulnerable to interference from many other electroactive substances.

## 4. Conclusions

The utilization of the new concept of a HRP-mediated capsaicin reaction for construction of a biosensor to determine capsaicin in chili fruits has been successful. The design of the biosensor via a photocuring technique yielded an amperometric biosensor that can determine capsaicin over a large linear range, showing good reproducibility, acceptable detection limits and satisfactory long term stability.

## Figures and Tables

**Table 1.** The recovery of spiked capsaicin determined in chili samples using biosensor.

**Added Capsaicin (μM)**	**Chili Extracted 5 h**			**Chili Extracted 15 min**		
	
	**Capsaicin Measured (μM)**	**Capsaicin Found (μM)**	**Recovery (%)**	**Capsaicin Measured (μM)**	**Capsaicin Found (μM)**	**Recovery (%)**
0	433 ± 45			430 ± 47		
401	832 ± 38	396	99	824 ± 93	394	98
601	1,041 ± 74	607	101	1,037 ± 132	607	101
801	1,208 ± 147	775	97	1,216 ± 108	786	98
1,001	1,445 ± 173	1,012	101	1,434 ± 59	1,004	100
1,201	1,648 ± 132	1,215	101	1,655 ± 168	1,225	102

Note: recovery (%) = (capsaicin found/added capsaicin) × 100; capsaicin measured: capsaicin concentration measured in chili samples with spiked capsaicin standards; capsaicin found: (capsaicin measured)-(capsaicin content of the non-spiked samples).
